# Two‐tone suppression between the ultrasounds above and within the hearing range in mice

**DOI:** 10.1113/EP092317

**Published:** 2025-02-23

**Authors:** Noriko Nagase, Hirokazu Kousaki, Bakushi Ogawa, Kazuhiro Horii, Iori Niitsu Morimoto, Chikara Abe, Takenori Ogawa, Fumiaki Nin

**Affiliations:** ^1^ Division of Biological Principles, Department of Physiology and Biophysics, Graduate School of Medicine Gifu University Gifu Japan; ^2^ Division of Sensorimotor Medicine, Department of Otolaryngology‐Head and Neck Surgery, Graduate School of Medicine Gifu University Gifu Japan; ^3^ Center for One Medicine Innovative Translational Research (COMIT) Gifu University Gifu Japan

**Keywords:** auditory brainstem response, cochlear microphonic potential, harmonics detection, ultrasonic hearing

## Abstract

Hearing range differs among various species. Ultrasound, which is audible to microbats and dolphins, is inaudible to humans through air conduction. However, it can create an auditory sensation when the stimulation is transmitted through the temporal bone. This phenomenon is known as ultrasonic hearing – sounds at frequencies exceeding the normal hearing range participate in audition. Mice are among the animals that possess one of the highest upper limits of the hearing range. Although ultrasonic hearing has been experimentally demonstrated in humans and guinea pigs, its existence in mice and interaction with ultrasound within the hearing range remain unknown. In this study, we found that ultrasound above the hearing range delivered through the temporal bone evokes the cochlear microphonic potential (CM) in mice. The CM synchronized with the applied single‐tone ultrasound, and was actively amplified. Furthermore, the amplitudes of the CM were suppressed by sound with subharmonic frequencies of the applied frequencies. The results indicate that hair cells in mice can detect ultrasound stimuli with frequencies over 120 kHz and ultrasounds within and above the hearing range evoked hair cell currents at the close position along the cochlea.

## Abstract (highlights)

- **What is the central question of this study?**
  Ultrasonic hearing has been experimentally demonstrated in humans and guinea pigs: does it exist in mice and if so, what is its interaction with ultrasound within the hearing range remain unknown.
- **What is the main finding and its importance?**
  Ultrasound beyond the hearing range evokes cochlear microphonic potentials and auditory brainstem responses in mice. Two‐tone suppression between ultrasounds within and above the hearing range suggests that nearby hair cells detect both the fundamental frequencies and their harmonics.

## 1 INTRODUCTION

In the cochlea, sound elicits a traveling wave in the sensory epithelium from the base towards the apex. The wave peaks at a frequency‐dependent location. Lower frequencies evoke a response on the cochlear apical epithelium, and high frequencies stimulate the basal epithelium (Bekesy, 1960; Gelfand, 2010; Hudspeth, 2013; Robles & Ruggero, 2001; Ulfendahl, 1997). This spatial arrangement of best frequencies is called tonotopy, and is thought to demarcate the hearing range (Liberman, 1982; Manley, 2017; Ruggero & Temchin, 2002). Elephants, for example, have an upper limit of approximately 6 kHz, while bats and dolphins can hear sounds up to more than 145 kHz (Echtler et al., 1994). Although both mice and guinea pigs possess hearing ranges extending into the ultrasound, the upper limit for mice is roughly one octave higher than that of guinea pigs. This difference can be explained by the anatomical variations in the pinna, tympanic membrane, basilar membrane and hair cells (Echtler et al., 1994; Rosowski, 1994).

In normal hearing, sound travels to the cochlea by way of the tympanic membrane and middle ear ossicles. This sound pathway is known as air conduction. In bone conduction, on the other hand, sounds directly engage the cochlea through the temporal bone (Bekesy, 1960; Gelfand, 2010). Ultrasound, defined as sound frequencies exceeding 20 kHz, is inaudible to humans. However, it can be perceived when it is presented through bone conduction (Corso, 1963; Gavreau, 1948; Haeff & Knox, 1963). For this phenomenon, called ultrasonic hearing, bone conduction was thought to be indispensable. While theoretical studies have demonstrated that bone‐conducted sound induces similar vibration patterns in the epithelium to those caused by air conduction (Tchumatchenko & Reichenbach, 2014), the reason for the perceptibility of bone‐conducted ultrasound above the hearing range has remained unclear.

**FIGURE 1:**
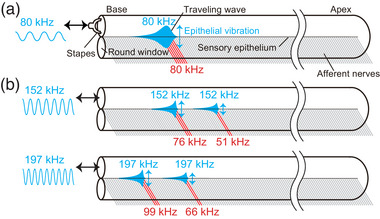
Depiction of single tone stimulus‐elicited cochlear traveling wave on the sensory epithelia. (a) Basic concept of cochlear tonotopy. Single tone stimulation excites epithelial vibrations at a particular position. In this and subsequent illustrations, the red lines indicate activated afferent nerves under stimulation. (b) Scheme of the epithelial vibrations based on harmonics detection under the single tone stimulus with the frequency above the hearing range. The frequency of the stimulus matches that of the vibration, but evokes multiple epithelial vibrations at different positions in subharmonic series of the fundamental frequency.

According to the classical model of cochlear tonotopy, hair cells at a specific position are activated by a traveling wave (Figure 1a). Recently, a study demonstrated that hair cells in the cochlear hook region detect ultrasound frequencies beyond the hearing range in guinea pigs (Horii et al., 2024). As a mechanism underlying this phenomenon, detection of harmonics by hair cells at the hook region has been reported. In this concept, hair cells detect the sounds not only at the fundamental frequency but also the frequencies of its harmonics, enabling hair cells positioned at geometric intervals to function cooperatively under single‐tone stimulation (Figure 1b). These findings indicate that the detectable frequency range of the cochlea is far broader than the canonical hearing range, which is traditionally defined by air conduction. In humans and guinea pigs, ultrasonic frequencies up to two octaves above the upper limit of their hearing range is detectable (Horii et al., 2024). However, the physiological mechanisms underlying ultrasonic hearing in mice, as well as its interaction with ultrasound within the hearing range, remain unclear.

In this study, we measured cochlear microphonic potentials (CMs) both within and above the hearing range in bone conduction. We also demonstrated that the CMs evoked by ultrasound above the hearing range interact with another tone within the hearing range. Additionally, we confirmed that mice sense ultrasound stimuli by demonstrating the presence of auditory brainstem response (ABR) to ultrasonic stimuli.

## 2 METHODS

### 2.1 Ethical approval

The animals were treated in compliance with the Guiding Principles for the Care and Use of Animals in the field of Physiological Science set by the Physiological Society of Japan. The experimental protocol was approved by the Institutional Animal Research Committee of Gifu University (Permission Number: 20220011). The experiments were carried out under the supervision of the committee and in accordance with the Guidelines for Animal Experiments of Gifu University and the Japanese Animal Protection and Management Law. C57BL/6JmsSlc mice (6–12 week of age; SLC Inc., Hamamatsu, Japan) were housed at the animal facility and kept under a 12‐h light–12‐h dark cycle (lights on 07.00–19.00 h) and given laboratory chow and water ad libitum. All animal handling and reporting complied with the Animal Research: Reporting of In Vivo Experiments (ARRIVE) guidelines.

### 2.2 Animals and experimental procedures

The study included 117 healthy male and female mice, each weighing 20–25 g. For CM and ABR measurements, the animals were anaesthetized with an intraperitoneal injection of a mixture of medetomidine (0.3 mg/kg), midazolam (4.0 mg/kg) and butorphanol (5.0 mg/kg). The animals breathed spontaneously without a respirator. In the CM recordings, the cochlea was exposed using a method similar to the one previously described (Horii et al., 2024). Body temperature was monitored with a thermometer and maintained by a heating pad. Supplemental doses of anaesthetic were administered to ensure areflexia to toe pinch. For *post mortem* CM recording, anesthetized animals were injected intraperitoneally with an overdose of urethane (1.5 g/kg). At the end of the experiment, the anaesthetized animals were decapitated.

Of the 117 animals included in this study, 78 were used for measurement of the CMs with single tone and two‐tone stimulations and 39 were used for measurement of the ABR. To ensure hearing sensitivity, we monitored the ABR thresholds both before and after the experiment. If the ABR thresholds changed by more than 30 dB at the frequency of 16 kHz, we eliminated the data from the analysis. Physiological responses were measured in approximately 13% of the preparations. The low success rate stemmed in part from the invasiveness and difficulty of middle ear surgery in mice. Transmeatal removal of the malleus–incus complex and perforation of the eardrum often led to decreased hearing sensitivity due to the development of a perilymphatic fistula. Of the 70 sensitive cochleae, 55 exhibited elevated ABR thresholds. From the 15 physiological cochleae, five cochleae were selected for each of the following measurements: ABR, CM with a single tone and CM with a two‐tone stimulus. For both ABR and CMs, the single‐tone group consisted of three males and two females, whereas for CMs with a two‐tone stimulus, the group included one male and four females.

### 2.3 Calibration of sound and ultrasound stimulation in bone conduction

We used a housed speaker (FT17H; Fostex, Tokyo, Japan) and piezoelectric actuators (PC4QM, PA44LEW; Thorlabs, Newton, NJ, USA) as stimulators, and they were powered using a function generator (WF1948; NF Corporation, Yokohama, Japan). For the calibration of pressure, we used an ultrasound microphone (Sokolich ultrasonic probe microphone system, CA, USA) and a high sensitivity hydrophone (TC4034, Teledyne Marine RESON, FL, USA) with an amplifier (EC6081 mk2, Teledyne Marine RESON). To maintain the compression force at 0.5 N on an object, we monitored the force between the stimulator and animal using a simple spring scale.

For air‐conducted sound stimulation, the tip of the speaker was inserted into the external ear canal, whereas the tip of the actuator was attached to the temporal bone for bone conduction. The basic aspects of bone‐conducted stimulation were similar to the one previously reported (Horii et al., 2024). To maintain the correct angle of the stimulator on the temporal bone, we extended the stimulator by attaching a cone‐shaped tip to the ceramic rod (Supporting information, Figure S1A), and inserted the tip into the external ear canal (Figure S1B). Since the bony canal wall is part of the temporal bone, we attached the tip of the stimulator to the bony wall. For two‐tone stimuli, we added another actuator to the mastoid bone (Figure S1C). To roughly compensate for the thresholds between air and bone conductions within the hearing range, we defined the standard bone compression pressure as 2 mPa and expressed all pressure levels in decibels throughout this study as previously reported (Horii et al., 2024). Additionally, to firmly attach the stimulator on the temporal bone, we inserted the tip into the bony canal wall for bone conduction (Figure S1B).

Due to the placement of the tip, the stimulator inevitably transmitted not only vibrations through the temporal bone but also sound through the tympanic membrane. To assess the effect of air conduction during this stimulation, we first recorded ABR without attaching the stimulator to the bony wall (Supporting information, Figure S2A). ABR thresholds were consistent with our data and reports on air conduction (Horii et al., 2024). After the removal of the tympanic membrane and malleus–incus complex, these thresholds clearly increased to inapplicable pressure levels (Figure S2B). Nevertheless, by placing the tip on the bony wall, thresholds returned to the level of normal conditions in air conduction. In addition, ABRs to the ultrasound above the hearing range became as prominent and the same as those in bone conduction (Figure S2C). For CM recording, the tympanic membrane and the malleus–incus complex were transmeatally removed using a Rosen needle commonly employed in otolaryngology. This procedure enabled direct cochlear stimulation via bone conduction using the stimulator, eliminating the influence of air conduction. For ABR recording, air‐conducted stimulation was first performed on intact mice, followed by the application of bone‐conducted ultrasound stimulation after the surgery.

### 2.4 Electrophysiological measurements

The basic method of CM recording was the same as described previously (Horii et al., 2024). Stainless steel electrodes were placed on the round window membrane and subcutaneously in the neck region. For the stimuli, 240 ms tone bursts at frequencies of 40, 80, 103, 152, 197 and 240 kHz with 20 ms rising and falling phases were generated by the function generator. To avoid artifact contamination, we implemented strict shielding of piezoelectric actuators. Local field potentials (LFPs) were amplified 5000‐fold and processed using an analog high‐pass filter (1 kHz) in a modified amplifier (Model 3000, A‐M Systems, Sequim, WA, USA). Acquired data were recorded by a digitizer with a sampling frequency of 2.4 MHz (PCIe‐6374; National Instruments, Austin, TX, USA) and eight sweeps were averaged using LabVIEW (LabVIEW 2019 SP2; National Instruments). We used a modified AC/DC amplifier (Horii et al., 2024). We defined the average amplitude of the recorded artifact as the limit of detection (LOD) in the CM recording. For stimuli in CM recordings of two‐tone interaction, the applied frequencies of the probe tone were 105, 140, 175 and 210 kHz, while the frequency of the suppressor tone was 70 kHz. For the ABR, the applied frequencies were 16, 40, 80 and 103 kHz in air‐conducted stimulation, and 16, 40, 80, 103, 152, 197 and 240 kHz in bone‐conduction. Individual signals emitted from the afferent auditory pathway were amplified 5000‐fold and processed with an analogue bandpass filter (300 Hz to 1 kHz) in a modified commercial amplifier. These data were digitally processed with a bandpass filter (300 Hz to 1 kHz), and 500 sweeps were averaged. ABR thresholds were defined as the lowest pressure level at which wave III was detected.

### 2.5 Statistical analysis

Means ± SD serve as descriptive statistics in Figures 3, 4, 4e and 5c, and all data are shown as a statistical summary. For multiple comparisons, two‐way ANOVA followed by Šidák's multiple comparisons test and one‐way ANOVA followed by Tukey's multiple comparisons test were performed for Figures 3, 4 (Prism 9; GraphPad Software, Boston, MA, USA). Data with a *P*‐value of <0.05 were considered statistically significant. All *n* values are indicated in the main text and the figures.

## 3 RESULTS

### 3.1 Cochlear microphonic potential evoked by ultrasonic stimulation in bone conduction

**FIGURE 2:**
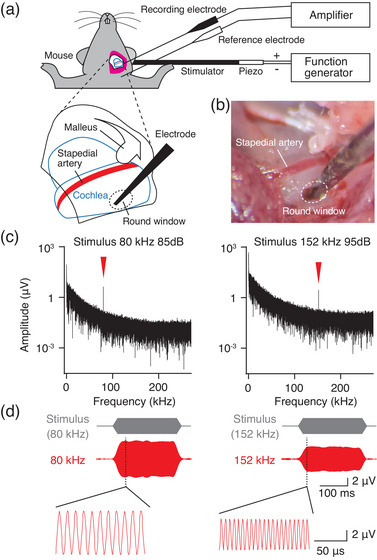
Recording of the local field potentials (LFP) in a mouse. (a) The experimental set‐up. A stimulator for the ultrasound stimulation was attached on the temporal bone, and a reference electrode was inserted into the subcutaneous tissue in the neck. (b) View and schematic representation of the middle ear cavity with the recording electrode. The left panel shows a schematic representation of the view in right panel. The tip of the recording electrode was placed on the round window. (c) Fourier amplitude spectra of the LFP during stimulation at 80 and 152 kHz at 85 and 95 dB in bone conduction. The red arrowheads demark the characteristic frequency peaks of the persistent electrical signals in the experiment. (d) LFP waveforms at 80 and 152 kHz with a schematic representation of the stimulus waveform (grey) in (c). A zoomed view of the LFP at 80 and 152 kHz under steady‐state stimulation is shown in the lower panels. Signals at 80 and 152 kHz are synchronized with the stimulus.

Ultrasound above the hearing range evoked CMs through bone conduction in guinea pigs (Horii et al., 2024). We therefore hypothesized that the hair cells in mouse cochlea are capable of initially transducing sound stimuli at higher frequencies than the audible range. To evaluate the functioning of the hair cells, we measured the CM, the alternating current component of the LFP generated by hair cells (Figure 2a) (Gelfand, 2010; Honrubia & Ward, 1968; Tasaki et al., 1952; Wever & Bray, 1930). To record the LFP, we placed a stainless steel electrode on the round window membrane (Figure 2b) and evaluated the frequency distribution of the LFP during stimulation. Figure 2c displays the fast Fourier transform of the voltage amplitude. When we delivered an 80 kHz, 85 dB stimulus to the temporal bone of the animal, a significant peak was visible at 80 kHz. Figure 2d exhibits the LFP waveforms with a stimulus waveform at the peaks. The waveform at 80 kHz was synchronized in time with the stimulus waveform. Therefore, the peak at 80 kHz within the hearing range was a CM. The applied frequency was next altered from 80 to 152 kHz. The peak was shifted to 152 kHz, but the waveform remained synchronous (Figure 2d). Based on the frequency dependence (Figure 2c,d), we defined the waveform at 152 kHz as the CM with a frequency above the hearing range. No harmonic distortion or electrical resonance was observed in the recording circuit for waveforms with frequencies below 270 kHz (Supporting information, Figure S3).

**FIGURE 3:**
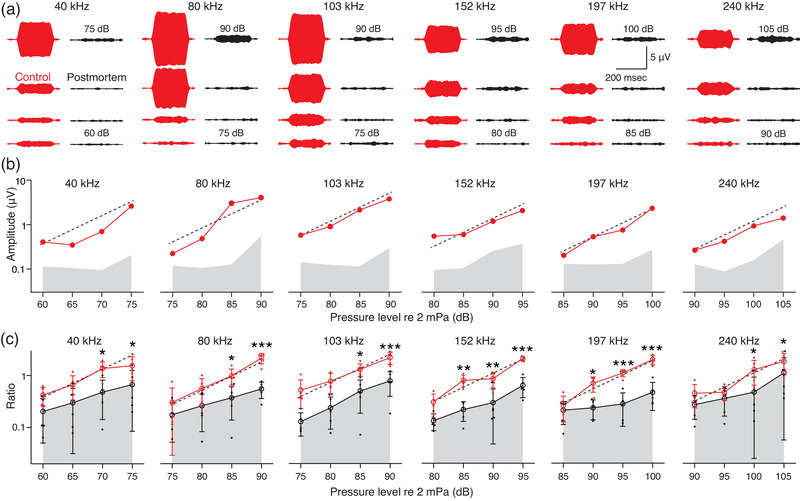
Recording cochlear microphonic potentials in bone conduction. (a) Representative trace of stimulation‐evoked CM waveforms in a male mouse. The results are plotted in 5 dB decrements. The left and right panels for each stimulus frequency show the control and *post mortem* conditions, respectively. (b) Summary profile of CM amplitude and its non‐linear response in the mouse. The level function relates to the amplitude of CM responses in the cochlea. Control data (red) during 40, and 80 kHz stimulation demonstrate compressive non‐linearity, whereas those during 103, 152, 197 and 240 kHz exhibit little non‐linear behaviour. The thin dashed lines indicate a linear relationship between the pressure level and CM amplitude. We defined the average amplitude of the recorded artifacts *post mortem* as the limit of detection (grey shaded area). The high‐resolution ultrasonic waveforms, with a sampling frequency of 2.4 MHz are down‐sampled to 24 kHz throughout this study. (c) Grouped CM amplitudes in five mice (*n* = 5). The continuous line and error bars indicate the means and standard deviations, respectively, for the control and *post mortem* conditions. The responses were normalized to the average values of the recorded voltages under control conditions and are shown as ratios. Asterisks represent a significant difference between normal and *post mortem* conditions (*P < 0.05, **P < 0.01, ***P < 0.001).

In the detection of sound within the hearing range, the active process of a healthy cochlea amplifies the CM while progressively reducing the enhancement of stronger stimuli (Tasaki et al., 1952). This level‐dependent amplification is known as compressive non‐linearity (Hudspeth, 2008), and is confirmed in ultrasonic hearing in guinea pigs (Horii et al., 2024). To determine whether a similar pattern is observed in mice, we next analysed the level function of the CM amplitude. The frequency and pressure level ranged from 40 to 240 kHz and from 60 to 105 dB, respectively (Figure 3a). Under physiological conditions, the amplitudes showed little non‐linearity at the frequencies of 40, 80, 103, 152, 197 and 240 kHz, but were significantly higher than those recorded under *post mortem* conditions shown by LOD (Figure 3b,c). Although the contribution of the active process seems to be subtle, the results indicate that the mouse hair cells are capable of responding to ultrasounds both within and beyond the hearing range. The latencies of the CMs were less than 0.002 ms at the frequencies of 152, 197 and 240 kHz (Bester et al., 2020), suggesting that ultrasound beyond the hearing range elicited a traveling wave at the hook region (Supporting information, Figure S4). These trends were confirmed in five animals (Figure 3c: *n* = 5).

### 3.2 Two‐tone suppression between the ultrasounds within and above the hearing range in CMs

In the classical model of cochlear frequency tuning, hair cells on the sensory epithelium are activated at a specific region along the cochlea turns by a single‐tone stimulus (Bekesy, 1960; Hudspeth, 2013; Robles & Ruggero, 2001). The active process of hair cells amplifies sound‐evoked epithelial vibrations through a feedback regulation mechanism, leading to characteristic phenomena such as otoacoustic emission and two‐tone suppression in multiple‐tone detection (Dong & Olson, 2016; Hudspeth, 2014). In two‐tone suppression, the cochlear responses to a probe tone are attenuated by the presence of a suppression tone. It is generally believed that two‐tone suppression occurs when the traveling waves evoked by probe and suppressor tones interact with each other (Dong & Olson, 2016; Geisler & Nuttall, 1997; Robles & Ruggero, 2001). In the previous study, we demonstrated both electrically and mechanically that hair cells in the hook region are resonant both to fundamental ultrasound and to its harmonics beyond the hearing range (Horii et al., 2024). This mechanism, called harmonic detection, supposes that simultaneous stimuli with harmonic series should elicit traveling waves with different frequencies in the hair cells at the close position along the cochlear turns. Although this assumption indicates that two‐tone suppression should occur between these ultrasounds, such experiments have not yet been demonstrated.

**FIGURE 4:**
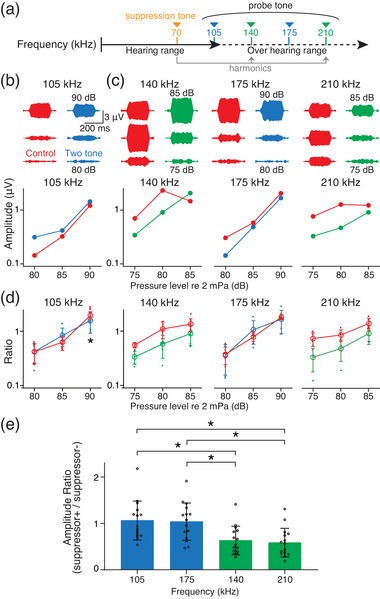
Effect of the suppression tone on CM evoked by probe tones. (a) Schematic drawings of the applied frequencies in two‐tone stimuli. Solid and dashed arrow exhibit the frequencies within and beyond the hearing range. Blue and green arrowheads indicate the probe tone, and orange arrowhead indicates the suppression tone. About 140 and 210 kHz are harmonics of 70 kHz. (b) Representative traces of evoked CM waveforms and summary profile of CM amplitudes under the probe tone of 105 kHz in a female mouse. The right and left waveforms show the CMs with and without the suppression tone of 70 kHz in upper panel, respectively. The results are plotted in 5 dB increments from 80 to 90 dB. (c) Representative traces of evoked CM waveforms and summary profile of CM amplitudes under the probe tone of 140, 175 and 210 kHz in the mouse. The results are plotted in 5 dB increments from 75 to 90 dB. The probe‐tone‐evoked CMs with 140 and 210 kHz are significantly suppressed by the suppressor, whereas those at 175 kHz are little affected. (d) Grouped CM amplitudes (*n* = 5). Responses were normalized to the averaged values of recorded voltages under control conditions and are shown as ratios. (e) Graph of the amplitude ratio of CMs with and without suppressor tone. The responses at all intensities were normalized by the amplitude under control conditions and are shown as ratios in each frequency (*n* = 15; collected from five animals). An asterisk represents a significant difference between groups (*P < 0.05).

Prior to the two‐tone suppression between the ultrasounds with harmonic series, we first measured the CM evoked by a probe tone of 105 kHz under the continuous suppression tone of 70 kHz within the hearing range (Figure 4a). For two‐tone stimuli, we attached two stimulators to the temporal bone simultaneously (Figure S1C). The frequency of the suppression tone was constant at 80 dB, while the pressure levels of the probe tone were variable. Similar to the previous studies of the two tone stimuli with frequencies that are numerically far apart (Cheatham & Dallos, 1982; Geisler et al., 1990; Robles & Ruggero, 2001), the suppression tone minimally changed the amplitudes of the CM evoked by the probe tone (Figure 4b). Next, we applied the probe tones with frequencies above the hearing range. The applied frequencies were 140, 175 and 210 kHz; 140 and 210 kHz are harmonics of the suppressor, while 175 kHz is anharmonic. In harmonic and anharmonic stimuli, the pressure level of the probe tones ranged from 75 to 85 dB, and 80 to 90 dB, respectively. The CM elicited by the probe tone of 175 kHz was little affected by the suppression tone as shown in the case of 105 kHz. In contrast, the CM amplitudes at the harmonic frequencies of 140 and 210 kHz were significantly decreased by the suppressor tone with the fundamental frequency at almost all intensities (Figure 4c). Collectively, the effects of the suppression tone with harmonic frequencies were significantly larger than those observed in anharmonic stimulation. These trends were confirmed in four additional animals (Figure 4d: *n* = 5), and effects of suppressor tone to harmonic probe tone in all intensities were significantly stronger than those to anharmonic probe tone (Figure 4e: *n* = 15, collected from five animals). The suppressor tones and the suppressor‐tone‐evoked CM did not contain harmonics and harmonic components, respectively (Supporting information, Figure S5). The result indicates that the probe‐tone‐ and suppression‐tone‐evoked hair cell currents at the close position along the cochlea, and the CMs share the source of hair cell mechano‐transduction current in the endolymph.

### 3.3 Auditory brainstem response in ultrasonic stimulation with frequency above the hearing range

**FIGURE 5:**
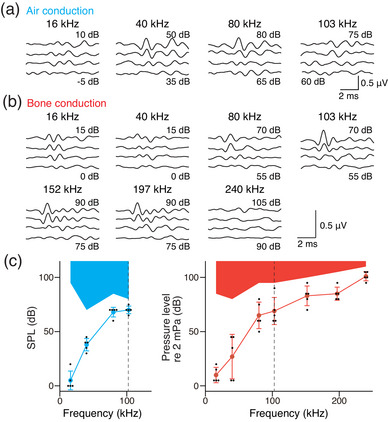
ABR signals and thresholds in air and bone conductions. (a) Representative examples of ABR signals under air‐conducted stimulation in a female mouse. The results are plotted in 5 dB decrements. (b) Representative examples of ABR signals under bone‐conducted stimulation in a male mouse. (c) Grouped ABR thresholds in air and bone stimulations. Left, and right panels show the data for air conduction (blue), and bone conduction (red), respectively (*n* = 5). Black dashed lines show the suspected upper limit of the hearing range; blue and red shaded areas indicate inapplicable pressure levels for each stimulation. ABR thresholds under air and bone‐conducted stimulations showed almost the same thresholds at audible frequencies, but ABR thresholds were observed at frequencies above the hearing range in bone conduction.

Ultrasounds above the animal's hearing range are detectable by humans and guinea pigs (Corso, 1963; Gavreau, 1948; Haeff & Knox, 1963; Horii et al., 2024; Yamashita et al., 2009, 2008). Our data in mice are consistent with these reports; ultrasound evokes CMs with frequencies above the hearing range. To confirm ultrasonic hearing in mice, we measured ABRs during air‐ and bone‐conducted sound stimulations (Figure 5a,b). Although ABR thresholds in both conductions were similar within the hearing range, ABR in bone conduction became prominent at intensities exceeding 80 dB for frequencies beyond the hearing range (Figure 5c, *n* = 5). Accordingly, mice can hear ultrasound above their hearing range, as previously reported for guinea pigs.

## 4 DISCUSSION

**FIGURE 6:**
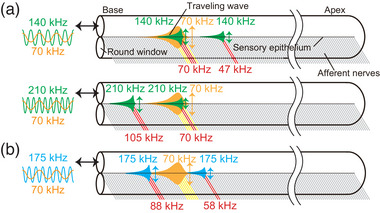
Depiction of a two‐tone stimulus eliciting a cochlear traveling wave on the sensory epithelia. (a) Scheme of the predicted epithelial vibrations of the two‐tone suppression in our study. The upper and lower panels show the epithelial vibrations under the probe tone stimulation at 140 and 210 kHz with the suppression tone stimulation at 70 kHz, respectively. Although the probe tone frequencies are numerically far from that of suppressor, evoked epithelial vibrations overlap at the position detecting the suppression tone under stimulation at 140 and 210 kHz. (b) The epithelial vibrations under the probe tone stimulation at 175 kHz with the suppression tone stimulation at 70 kHz. All epithelial vibrations are independent in stimulations at 175 kHz with 70 kHz suppression.

In this study, we measured CMs synchronized with ultrasound frequencies beyond the mouse hearing range, and confirmed that ultrasounds evoked ABR. Although CMs as a function of intensity exhibited non‐linearity in guinea pigs, CMs in mice showed an almost linear relationship. In contrast, CMs are actively amplified in both guinea pigs and mice. How can the sensory epithelium transduce the ultrasounds into CMs above the hearing range? Recently, detection of harmonics by hair cells at the hook region has been reported in guinea pigs (Horii et al., 2024); hair cells spaced at geometric intervals collaborate under single‐tone stimulation (Figure 1b). This concept is supported by our result from two‐tone interaction experiments. In two‐tone interactions, CMs evoked by stimulation with different tones influence each other, and the magnitude of this influence is dependent on the frequency of the tones (Robles & Ruggero, 2001). When the frequency difference is less than 10%, as in the case where the probe tone frequency is near that of the suppressor, CM amplitudes are suppressed due to the overlap of the evoked epithelial vibrations. In contrast, when the difference is more than one octave, little interaction has been observed (Cheatham & Dallos, 1982; Geisler et al., 1990; Robles & Ruggero, 2001). This phenomenon is observed in auditory nerve, hair cells and basilar membrane (Cheatham & Dallos, 1992; Cooper, 1996; Dong & Olson, 2016; Galambos & Davis, 1944; Geisler & Nuttall, 1997; Geisler et al., 1990; Nomoto et al., 1964; Patuzzi et al., 1984; Rhode & Cooper, 1993; Ruggero et al., 1992; Sachs & Kiang, 1968). In this study, although the frequencies of 140 and 210 kHz were more than one octave higher than that of the suppressor, evoked CMs were significantly decreased (Figure 4d). This intrinsic phenomenon can be explained by the epithelial vibrations induced by detection of harmonics. Since ultrasound over the hearing range should elicit the epithelial vibration in the region detecting the fundamental frequency, the epithelial vibration overlaps the vibration induced by the subharmonic suppressor tone (Figures 4a and 6a). This overlap results in the simultaneous activation of hair cells by both tones, generating two MET currents with different frequencies. These currents summate within the endolymph, flowing through the cochlear lateral wall, and ultimately suppressing CMs (Dong & Olson, 2016; Geisler et al., 1990; Nin et al., 2012, 2017). In contrast, the epithelial vibration does not interact with the vibration induced by the non‐subharmonic suppressor tone (Figure 6b). The suppression between sounds with harmonic series could ubiquitously occur in the cochlea. Further experiments on multiple tone suppression by harmonic sounds within the hearing range should be performed in vivo.

It is well‐known that the bandwidth of the hearing range is crucial for animal survival. For communication, humans or birds perceive subtle modulated frequency of voices or chirps with high frequency discrimination. In bats and dolphins, ultrasound is used not only for communication but also for echolocation. In echolocation, animals recognize millisecond‐scale time lag of the reflected click sound from an object with high time resolution (Corcoran & Moss, 2017; Yovel et al., 2011). The waveform of a click is mathematically divided into frequencies with a harmonic series. These harmonics could be received by the hair cells covering the fundamental frequency in the hook region. In this harmonics detection, although the frequency discrimination is lower than that in the reception of fundamental frequency (Horii et al., 2024; Yamashita et al., 2009, 2008), the time resolution should be maintained. This speculation indicates that the boundaries of tonotopy do not inevitably demarcate the limit of the hearing range. To understand the relationship between these boundaries, further physiological studies based on anatomy may be required.

## AUTHOR CONTRIBUTIONS

Noriko Nagase, Data curation, Formal analysis, Investigation, Visualization, Methodology, Writing—original draft,; Hirokazu Kousaki, Data curation, Formal analysis; Bakushi Ogawa, Data curation, Formal analysis; Kazuhiro Horii, Data curation, Supervision, Funding acquisition, Investigation, Methodology, Project administration, Writing—original draft; Iori Niitsu Morimoto, Data curation, Formal analysis, Investigation; Chikara Abe, Formal analysis; Takenori Ogawa, Resources, Supervision; Fumiaki Nin, Conceptualization, Data curation, Formal analysis, Investigation, Visualization, Methodology, Project administration, Supervision, Resources, Funding Acquisition, Writing—original draft, Wrigint—review and editing. All authors have read and approved the final version of this manuscript and agree to be accountable for all aspects of the work in ensuring that questions related to the accuracy or integrity of any part of the work are appropriately investigated and resolved. All persons designated as authors qualify for authorship, and all those who qualify for authorship are listed.

## CONFLICT OF INTEREST

The authors declare no conflict of interest.

## Supporting information



Figures S1–S5.

Data for Materials and Methods, Figures 2, 3 and 4.

## Data Availability

All data supporting the findings in this study are available within the paper, Supplementary Information and figshare (https://doi.org/10.6084/m9.figshare.26867224).
