# Supplementary material for: Two‐tone suppression between the ultrasounds above and within the hearing range in mice
Source: Exp Physiol. 2025 Feb 23;110(8):1129–37. doi: 10.1113/EP092317 (PMC12314633; doi:10.1113/EP092317)
Supplement: Supplementary file 1 — Figures S1–S5. [file EPH-110-1129-s002.pdf]

Supplementary information for

Two-tone suppression between the ultrasounds above and within the hearing range in mice

Noriko Nagase[a, b], Hirokazu Kousaki[a, b], Bakushi Ogawa[a,b], Kazuhiro Horii[a], Iori Niitsu, Morimoto[a], Chikara Abe[a], Takenori Ogawa[b], Fumiaki Nin[a,c,1].

a) Division of Biological Principles, Department of Physiology and Biophysics, Graduate School of Medicine, Gifu University, Gifu, Japan 501-1194.

b) Division of Sensorimotor Medicine, Department of Otolaryngology-Head and Neck Surgery, Graduate School of Medicine, Gifu University, Gifu, Japan 501-1194.

c) Center for One Medicine Innovative Translational Research (COMIT), Gifu University, Gifu, Japan 501-1194.

[1]To whom correspondence may be addressed. Email: nin.fumiaki.u7@f.gifu-u.ac.jp

*Fumiaki Nin.

Email: nin.fumiaki.u7@f.gifu-u.ac.jp

This PDF file includes:

Figures **S1** to **S5**

Figures

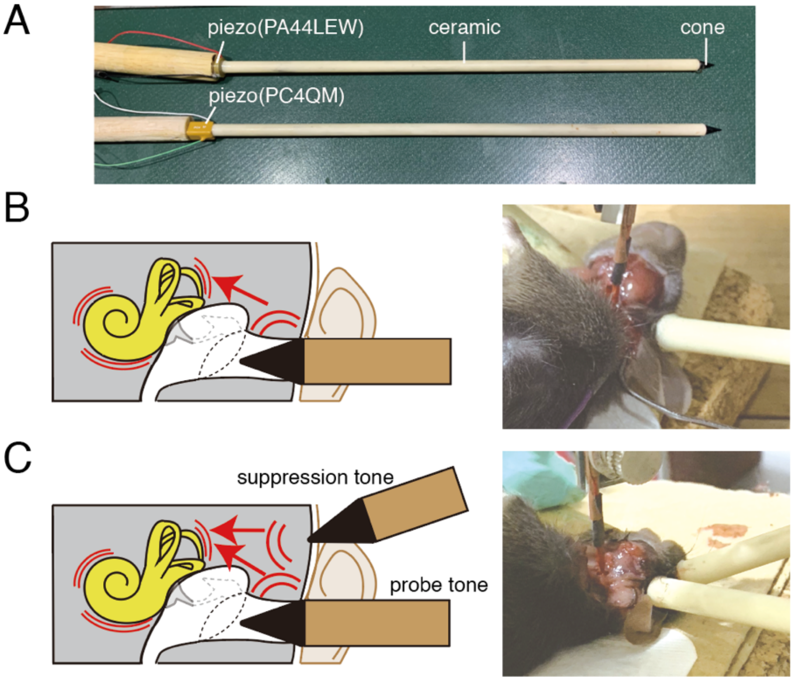

Figure S1. Single and two tone stimulations in experiments. (A) A view of the stimulators. The tip of a ceramic rod was connected to a piezo actuator, while the other tip of the rod was attached to a cone. (B, C) Schematic diagrams and views of bone conduction in single and two tone stimulation. In both stimuli, bone-conducted vibration directly reaches the cochlea through the temporal bone (red arrow). To avoid contamination of air-conducted sound, the tympanic membrane and malleus-incus complex were surgically removed.

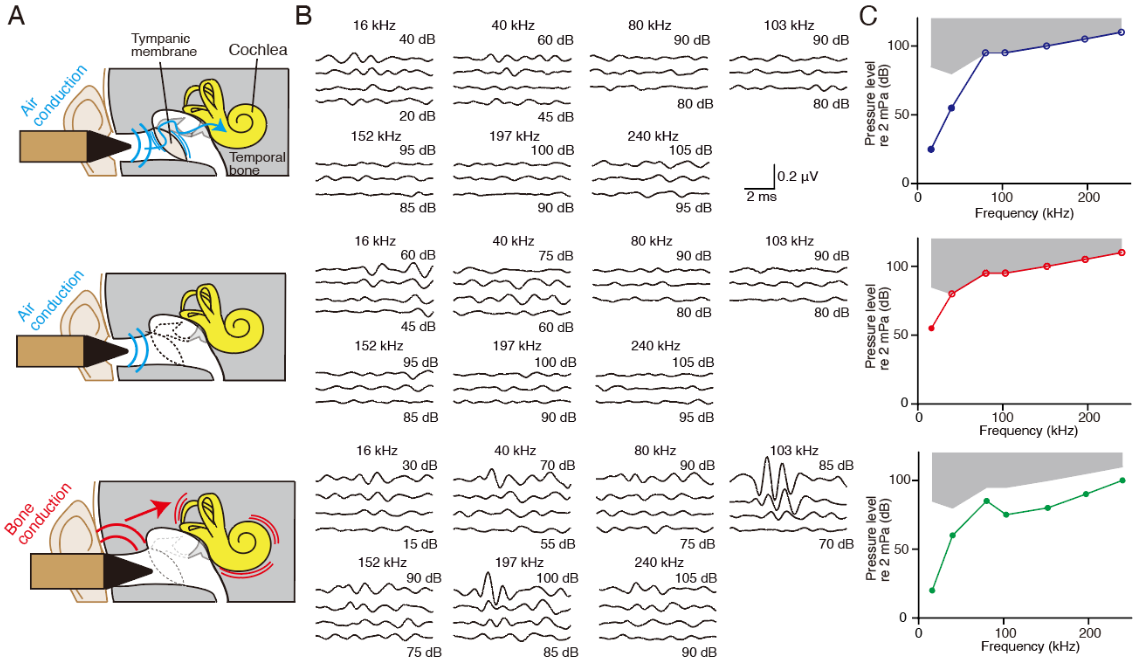

Figure S2. ABR thresholds air and bone conductions by different coordinate of the stimulator in a mouse. (A) Schematic diagrams of air and bone conductions through the stimulator. In this figure, upper and middle panels show air conduction with and without the tympanic membrane and malleus-incus complex, while lower panel shows bone conduction through a bony canal wall. (B, C) Representative ABR signals and thresholds in a mouse. Upper and lower traces exhibit control and post-exposure conditions. Grey shaded areas indicate inapplicable pressure levels for each stimulation. ABR thresholds of 16 and 40 kHz under air-conducted stimulation with intact middle ear showed almost the same thresholds in Figure 4C. After the removal of the tympanic membrane and malleus-incus complex, the thresholds increased to inapplicable pressure levels. However, when the tip of the stimulator was attached to a bony canal wall, ABR thresholds were observed in applicable pressure levels at both frequencies within and beyond the hearing range through bone conduction.

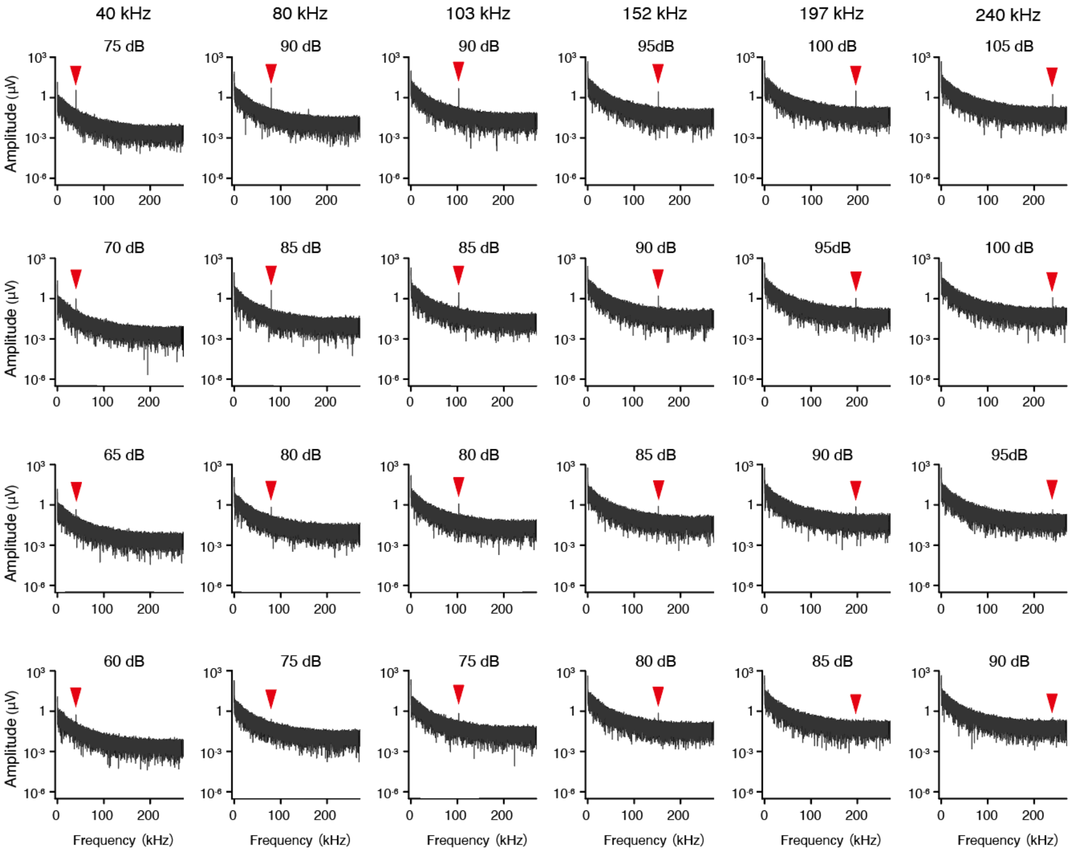

Figure S3. Fourier amplitude spectra of the LFP in a mouse. Fourier amplitude spectrum of the LFP in bone conduction. The arrowheads demark the frequency peaks of persistent electrical signals. Red filled arrowheads indicate stimulus frequencies. In all stimulations, harmonic peaks were not observed except for the strongest stimulation at 80 kHz.

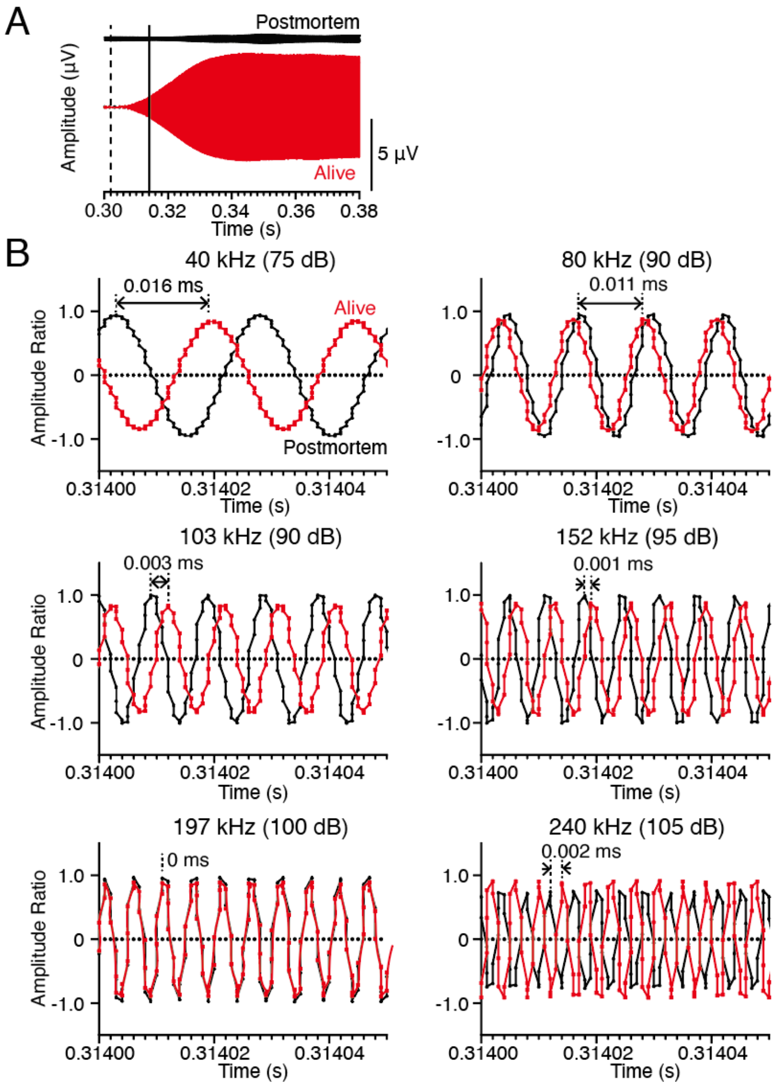

Figure S4. Latencies of physiological cochlear microphonic potentials under high-intensity ultrasound stimulation. (A) Representative CM waveforms under alive and postmortem conditions in the mouse presented in Figure 3. Dashed and solid lines indicate the periods for reference and analysis, respectively. (B) Enlarged CM waveforms from the analysed region. To ensure accurate comparison, the waveform phases were compensated using pre-stimulus baseline data under both conditions, and the amplitudes were standardized. Across all frequencies, the CM latencies between the reference and analysis periods were less than one cycle of the stimulus.

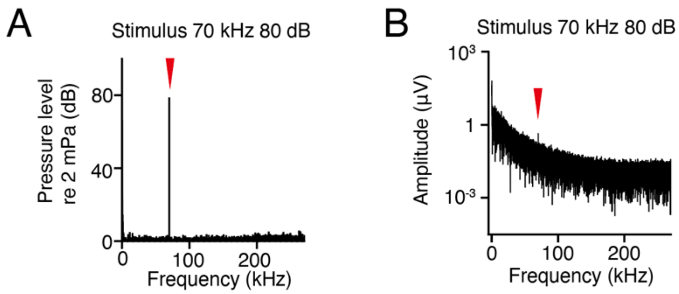

Figure S5. Fourier amplitude spectra of the suppression tone and LFP in a mouse. (A) Fourier amplitude spectra of the suppression tone in the stimulus of 70 kHz at 80 dB. Red arrowhead demarks the frequency peaks of the tone recorded by a hydrophone. Harmonic peaks were not observed. (B) Fourier amplitude spectra of LFP in the stimulus of 70 kHz at 80 dB. Harmonic peaks were not observed.
